# Mental Health and the Perceived Usability of Digital Mental Health Tools Among Essential Workers and People Unemployed Due to COVID-19: Cross-sectional Survey Study

**DOI:** 10.2196/28360

**Published:** 2021-08-05

**Authors:** Felicia Mata-Greve, Morgan Johnson, Michael D Pullmann, Emily C Friedman, Isabell Griffith Fillipo, Katherine A Comtois, Patricia Arean

**Affiliations:** 1 Department of Psychiatry and Behavioral Sciences University of Washington Seattle, WA United States; 2 Advanced Laboratories for Accelerating the Reach and Impact of Treatments for Youth and Adults with Mental Illness (ALACRITY) Center University of Washington Seattle, WA United States; 3 Creating Technological Innovations (CREATIV) Lab University of Washington Seattle, WA United States; 4 Center for Suicide Prevention and Recovery University of Washington Seattle, WA United States

**Keywords:** digital health, COVID-19, essential worker, unemployed, usability, user burden, mental health, e-mental health, survey, distress

## Abstract

**Background:**

COVID-19 has created serious mental health consequences for essential workers or people who have become unemployed as a result of the pandemic. Digital mental health tools have the potential to address this problem in a timely and efficient manner.

**Objective:**

The purpose of this study was to document the extent of digital mental health tool (DMHT) use by essential workers and those unemployed due to COVID-19, including asking participants to rate the usability and user burden of the DMHT they used most to cope. We also explored which aspects and features of DMHTs were seen as necessary for managing stress during a pandemic by having participants design their own ideal DMHT.

**Methods:**

A total of 2000 people were recruited from an online research community (Prolific) to complete a one-time survey about mental health symptoms, DMHT use, and preferred digital mental health features.

**Results:**

The final sample included 1987 US residents that identified as either an essential worker or someone who was unemployed due to COVID-19. Almost three-quarters of the sample (1479/1987, 74.8%) reported clinically significant emotional distress. Only 14.2% (277/1957) of the sample used a DMHT to cope with stress associated with COVID-19. Of those who used DMHTs to cope with COVID-19, meditation apps were the most common (119/261, 45.6%). Usability was broadly in the acceptable range, although participants unemployed due to COVID-19 were less likely to report user burden with DMHTs than essential workers (*t*_198.1_=–3.89, *P*<.001). Individuals with emotional distress reported higher financial burden for their DMHT than nondistressed individuals (*t*_69.0_=–3.21, *P*=.01). When the sample was provided the option to build their own DMHT, the most desired features were a combination of mindfulness/meditation (1271/1987, 64.0%), information or education (1254/1987, 63.1%), distraction tools (1170/1987, 58.9%), symptom tracking for mood and sleep (1160/1987, 58.4%), link to mental health resources (1140/1987, 57.4%), and positive psychology (1131/1986, 56.9%). Subgroups by employment, distress, and previous DMHT use status had varied preferences. Of those who did not use a DMHT to cope with COVID-19, most indicated that they did not consider looking for such a tool to help with coping (1179/1710, 68.9%).

**Conclusions:**

Despite the potential need for DMHTs, this study found that the use of such tools remains similar to prepandemic levels. This study also found that regardless of the level of distress or even past experience using an app to cope with COVID-19, it is possible to develop a COVID-19 coping app that would appeal to a majority of essential workers and unemployed persons.

## Introduction

### Background

The COVID-19 pandemic has led to necessary public health mandates, such as physical distancing and stay-at-home orders. While these orders are important to contain the outbreak, they have led to concerns about increased isolation and loneliness among the general population, and prolonged exposure to stress among essential workers (eg, those working in food distribution, construction, mail delivery, etc) and those who are unemployed or furloughed owing to the pandemic [1-4]. Rates of negative mental health outcomes, especially fear, anxiety, and stress, in the general population during this pandemic are higher compared to prepandemic times [1,5].

Individuals struggling financially are reporting challenges with job security (ie, being laid off), housing costs, and making enough money to make ends meet [6]. Essential workers and those unemployed due to COVID-19 have many unique stressors, including but not limited to, concern about COVID-19 exposure, caring for family while working or searching for work, uncertainty about their job security, financial stress, guilt about not contributing to frontline COVID-19 efforts, under- or uninsured status, and access to no or nonmedical grade personal protective equipment [1-4]. While both groups have shared concerns, recent studies have shown that half of all essential workers are likely experiencing at least one adverse mental health symptom and increased anxiety or fatigue due to work demands in high stress or changing settings [3,7]. For the unemployed, there is concern about higher rates of suicidality and suicide attempts. Previous pandemics, such as the Spanish flu of 1918 and the 2003 SARS (severe acute respiratory syndrome) epidemic, led to an increase in suicide, and loss of employment and financial stress are risk factors for suicide [4,8]. Although the recent availability of vaccines and the eventual reopening of services mean that these concerns will eventually resolve, the need to understand how to best support essential workers and unemployed people emotionally during this time is still important, as future pandemics are predicted to be likely [9], and the long-term emotional impact of the current pandemic is still unknown [10].

In response to these mental health concerns, public health systems and digital mental health companies responded by increasing access to existing technology-based care (ie, telemedicine) or modifying digital mental health tools (DMHTs), such as online resources or mobile phone apps to address perceived concerns specific to COVID-19. For example, in the United States, Medicare restrictions on telemedicine were lifted to allow for better access to health care [11]. DMHTs are also available as potential solutions to decrease stress and mental health symptoms and address the mental health care shortage during COVID-19 [8,12]. In anticipation of the need for low- or no-cost care, organizations such as the Veterans Affairs Health Care System created a free mobile app to help veterans cope with COVID-19. A report from March 2020, as physical distancing began in the United States, found that there was an increased volume of people using these tools [13]. In addition, many organizations and tech companies are turning to DMHTs to support the emotional well-being of frontline health care workers [14].

These recent events lend an important opportunity to learn about the utility of digital mental health to support populations impacted by prolonged pandemic conditions. No research has evaluated the use of DMHTs by two of the most affected populations outside of frontline health care workers and older adults or adults with disability: essential workers and those unemployed due to COVID-19. As identified in several studies, the use of DMHTs tends to be poor, with most people downloading then discontinuing use of these tools in quick succession [15,16]. As Mohr and colleagues [17] have noted, digital mental health service use could be improved if intervention developers better understood what features people felt were important to have, the usability of these tools, and what role these services should have in the context of mental wellness [18-20].

### This Study

Considering the need to better understand the mental health challenges faced by essential workers and those unemployed due to COVID-19, the potential long-term effects of the societal challenges imposed by the pandemic, the potential for future pandemics, and the limited information we have on the usability and user burden of DMHTs to cope with the stress of COVID-19, we conducted a study with the following aims:

- Aim 1: Document psychological distress through clinically validated measures by the total sample, employment status (ie, unemployed due to COVID-19 and essential workers), and DMHT use (ie, reported using DMHTs to cope with COVID-19, reported not using DMHTs to cope with COVID-19);
- Aim 2: Explore DMHT use in response to COVID-19–related stress and differences by employment status and psychological distress (ie, distressed, not distressed);
- Aim 3: Assess usability and user burden ratings of DMHTs by total sample, employment status, and psychological distress;
- Aim 4: Understand the needs of these at-risk populations by identifying what DMHT features were ranked as most important by employment status, psychological distress, and DMHT use during this time.

## Methods

### Recruitment

A total of 2000 adults (≥18 years old) were recruited from Prolific Research Platform [21]. Using online research platforms is becoming increasingly popular in behavioral health research due its affordability, efficiency, access, and reliability [22]. Recent studies highlight that participants recruited from Prolific are more diverse and honest as well as provide higher data quality compared to other popular platforms, such as Amazon Mechanical Turk [22,23]. This national, cross-sectional study collected responses from October 26, 2020, to December 14, 2020. Participants were screened and invited to consent for participation in the anonymous, confidential survey online. Each participant was paid $3. The research was approved by the University of Washington’s institutional review board.

### Measures

Measures were selected and created to maximize participant engagement and reduce respondent burden. The investigative team reviewed brief measures of constructs of interest and gave preference to longer measures where no reliable or valid brief measure was available.

#### Inclusion Screening

Participants must have been ≥18 years old, speak English, and self-reported as either an essential worker during COVID-19 or unemployed or furloughed due to COVID-19. They also had the opportunity to indicate their current job (if an essential worker) or past job (if an unemployed worker). Participants were excluded if they were under 18 years of age, did not speak English, had no access to a mobile device (eg, smartphone or tablet), did not report being an essential worker or unemployed due to COVID-19, or lived outside of the United States.

#### Bad-Actor Screening

Even with the best safeguards in place, online recruitment can sometimes result in the accidental inclusion of individuals who participate in bad faith to accumulate monetary incentives (“bad actors”) [24]. We instituted the procedures explained below to identify potential bad actors.

The first was to use research platforms (described above) that conduct their own extensive participant vetting. These procedures include but are not limited to: (1) every account needing a unique non-VOIP (voice over IP) phone number to verify, (2) restricting signups based on IP address and internet service provider, (3) limiting the number of accounts that can use the same IP address and machine to prevent duplicate accounts, (4) limiting the number of unique IP addresses per study, and (5) unique payment accounts (eg, PayPal) for each participant account. For example, in order to have 2 participant accounts that receive payment from Prolific, a participant would need to have 2 PayPal accounts. Payment accounts, such as PayPal, have steps to prevent duplicate accounts, such as analyzing internal data to monitor for patterns of unusual use [25].

The second method involved the use of an attention check built into our survey [26]. This method consisted of one question where participants were given this instruction: “To confirm you are paying attention, please select ‘strongly disagree’” and then choices between strongly agree to strongly disagree were provided.

The third method involved the review of open-ended responses to screen out bot-like communication, repetitious, and nonsensical responses. Each of these methods confirmed that the final sample in this study could be qualified as comprising “good actors.”

#### Demographics

Participants completed a questionnaire about demographics, which collected information about age, race, ethnicity, gender identity, sexual orientation, marital status, education, employment status, income, and living situation.

#### Mental Health and Possible Substance Use Disorder

Participants completed the 2-item Patient Health Questionnaire (PHQ-2) [27], the 2-item Generalized Anxiety Disorder (GAD-2) [28], and the Cut-Annoyed-Guilty-Eye Adapted to Include Drugs (CAGE-AID) [29]. The PHQ-2 and GAD-2 have good sensitivity and specificity with sensitivity to change over time in comparison to the PHQ-9 and GAD-7 [28-30]. The CAGE-AID demonstrates good sensitivity and poor specificity for substance use disorders. As a result, individuals who scored beyond the cut-off on the CAGE-AID (≥1) were categorized as a possible case of substance use disorder, in accordance with the National HIV Curriculum [29,31].

#### Suicidal Behaviors

Suicidal behaviors were measured using the Suicide Behaviors Questionnaire–Revised (SBQ-R) [32], a 4-item self-report measure that assesses suicide attempts, ideation, communication, and intent in one’s lifetime. If the total score is greater than or equal to 7, the score is deemed to have good sensitivity and specificity for identifying individuals at risk for suicidal behaviors in a nonpsychiatric general adult population. Given some limitations of the SBQ-R, a single validated item (ie, “Have you attempted to kill yourself?”) was added. The addition of this item provides further accuracy and classification of individuals at risk of suicide [33].

#### Psychological Distress

Participants were placed in the “distressed” category if they endorsed one or more of the clinical cut-offs, which included ≥3 on the PHQ-2 [27], ≥3 on the GAD-2 [28], ≥1 on the CAGE-AID, ≥7 [29,31] on SBQ-R [32], or reported a history of a suicide attempt [33].

#### DMHT Questionnaire

This questionnaire was developed by the research team with expertise in digital mental health (author PA). The measure was tested for face validity, understandability, and respondent burden among the internal group. The questionnaire consisted of three distinct tasks: use of DMHTs during COVID-19, usability and burden of DMHTs during COVID-19, and design of an ideal DMHT for COVID-19, which are described below.

##### Use of DMHTs

**Figure 1:**
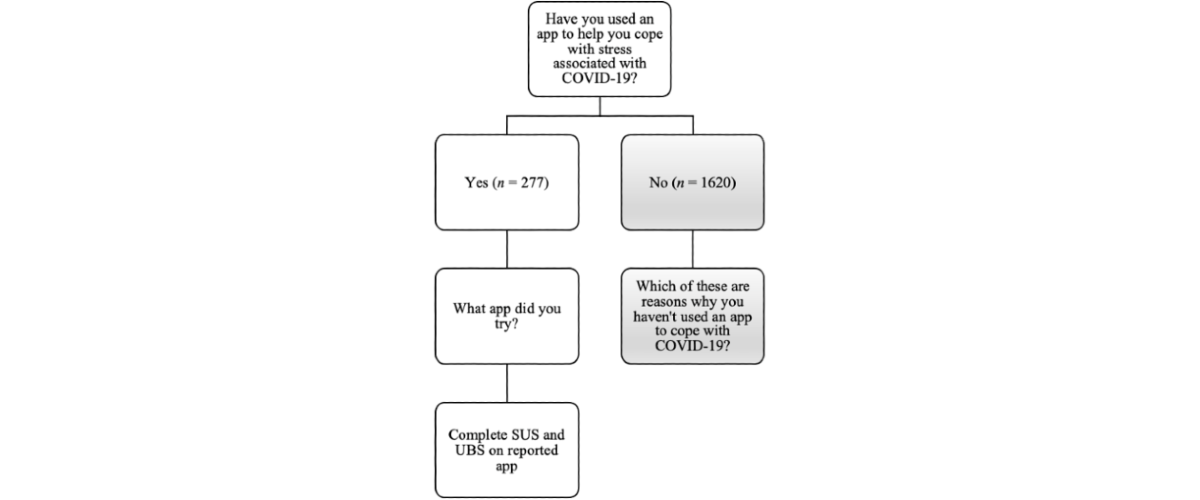
Respondent pathway. SUS: System Usability Scale, UBS: Use Burden Scale.

All participants were asked whether they have used an app to cope with stress associated with COVID-19. If the participant responded yes, they were asked to list which apps they used, and if they used more than one, to list the app they used the most to cope with COVID-19. Participants were then asked to rate the app that they used most frequently in terms of features they liked, features they did not like, and then on the app’s usability and user burden. If participants did not report using an app to cope with COVID-19 stress, they were asked to provide reasons for why they did not use an app (Figure 1).

##### Usability

Usability was measured with the System Usability Scale (SUS) [34], a 10-item measure that examines the usability of a particular intervention. The scale assesses a system’s likability, learnability, complexity, need for technical support, system integration, and efficiency. The SUS is the industry standard for measuring the usability of a variety of digital tools and systems and has normative data to allow for cross system and app comparisons, even between those that are outwardly very dissimilar to one another [35].

##### User Burden

User burden was measured using the 20-item Use Burden Scale (UBS) [36]. This scale creates five subscales to assess different types of user burden: difficulty of use (“this app demands too much mental effort”), physical demands (“use of this app is too physically demanding”), time and social burden (“I spend too much time using this app”; “using this app has a negative impact on my social life”), mental and emotional burden (“this app presents too much information at once”), and privacy and financial burden (“the value of the app is not worth the cost for me”). This measure was developed in order to assess the adoption, retention, and experience of various technologies with the ability to compare and calibrate burden across different tools. User burden is linked to app retention and has been used in the context of mobile app research [37].

##### Design of a COVID-19 App

All participants, regardless of whether they reported app use for stress associated with COVID-19, were asked which features they thought would be helpful to include in an app for coping with COVID-19 (ie, information or education, meditation/mindfulness, symptom tracking, brain games, distraction tools, gratitude exercises, links to resources, chatbot, or tips to cope with COVID-19) on a scale from 0=“not at all important” to 9=“very important.” This method of asking opinions of those who do and do not use digital technology, particularly when the needs of a given population are unknown, is commonly used in app development. The opinions of people familiar and unfamiliar with apps are needed to design a digital tool with the broadest reach [38].

After indicating which features participants preferred in an app to cope with COVID-19, they were then asked to build their own app, by selecting from a preset list of features and then adding their own desired features that were not previously listed. The app feature list was created using premade categories from One Mind Psyberguide [39], a nonprofit tool that reviews digital mental health tools for consumers, and M-Health Index and Navigation Database (MIND) [40] (see Multimedia Appendix 1 for the full survey).

### Statistical Analysis

To describe the sample, we ran crosstabulations (with chi-square tests or Fisher exact tests) and independent samples *t* tests to examine possible differences in the demographic and descriptive variables by employment status (ie, unemployed vs essential worker groups) and DMHT use (ie, DMHT user vs non–DMHT user). For variables with multiple discrete categories (eg, education), if these analyses indicated a significant omnibus chi-square test, we examined standardized residuals to identify which categories were responsible for the omnibus significant difference, and reported on all categories with absolute value standardized residuals greater than 2.

For the first aim, descriptive statistics were used to document the frequencies and means of the psychological distress composite among the entire sample and stratified by employment status. We also compared those who reported using an app to cope with COVID-19 to those who reported not using an app to cope with COVID-19. Specific reports on depression, anxiety, possible substance use disorder, suicidal behavior, and history of suicide attempt may be found in Multimedia Appendix 2.

For the second aim, we calculated frequencies and differences in DMHT use for the whole sample, between essential workers and those unemployed and between those reporting distress and no distress.

For the third aim, we computed means and SDs to examine DMHT ratings from the SUS and the UBS only for those who reported using a DMHT to cope with COVID-19. Differences across the top 3 apps were assessed using an ANOVA (analysis of variance). For the sample that did not report using a DMHT to cope during COVID-19, we provided the reasons for not using a DMHT and the frequency by which those reasons were endorsed in the sample.

For the fourth aim, we computed frequencies and central tendencies of the data to assess preferred DMHT components for the whole sample and compared these findings first between essential workers and those unemployed, then between distressed and nondistressed subsamples, and finally between those who reported having used a DMHT and those who did not.

The aims described above that examined significant differences by employment, distress, or DMHT use status were assessed using chi-square tests, Fisher exact tests, or independent samples *t* tests. All statistical analyses were performed with SAS version 9.4 (SAS Institute Inc). To adjust for increased type 1 error rates due to multiple tests, we applied the Benjamini-Hochberg procedure, which applies the acceptable fraction of tests that may be erroneously statistically significant, deemed the “false discovery rate” [41,42]. We applied a false discovery rate (Q) of 10% to 119 statistical tests.

Open-ended responses from the DMHT survey for categories (ie, “What app did you try? If you tried more than one app, please pick the one you liked the most”) and app features listed during the create-your-own-app survey were qualitatively coded. Like Rubanovich et al [43], the first author (FM-G) referenced the Apple App Store and Google Play to verify spelling and DMHT titles. As an example, *Calm*, *CALM*, *Calm App*, *Calm*, and *Camh* were all coded as “Calm.” If a DMHT was unable to be identified via Google Play, Apple App Store, or an internet search, or the participant response was undecipherable (eg, “IDK,” “NA”), it was categorized as missing (n=18).

Categorization of DMHTs was completed by authors FM-G and MJ. Informed by a modified grounded theory approach [44], each response was reviewed in order to identify meaningful units of information. Responses were compared with one another and grouped based on common responses until categories were identified. If the authors were unfamiliar with a DMHT, they read descriptions and reviews of the DMHT to determine its main feature. Some participants described DMHTs instead of names. In these cases, the response was coded for a DMHT category, but not for a specific DMHT title. As an example, the following responses, “I used a few meditation apps and one about CBT,” “mindfulness app,” and “meditation app” were coded into the mindfulness/meditation category. Categories and definitions were informed by Psyberguide, MIND, and experience working with digital mental health researchers. An identical process was conducted to code desired app features.

### Data Exclusion and Cleaning

Duplicate cases were identified and removed. Missingness accounted for less than 5% of the data evaluated item by item. Measures were scored unless all items were missing. As an exception, PHQ-2, GAD-2, and CAGE-AID required all items to be answered to attain a final score.

## Results

### Sample Description

A total of 2485 participants completed the initial screener. Of this, 598 (23.7%) observations were deleted due to missing IDs, duplicate responses, “bad actors,” or not meeting inclusion criteria. The final analytic sample (Table 1) consisted of 1987 adults with 1013 (50.9%) participants reporting unemployment due to COVID-19 and 974 (49.0%) identifying as an essential worker during COVID-19. The most common open-ended responses for jobs among essential workers included education, customer service or retail, management, information technology (IT), health care, pharmacy, delivery or postal work, and food service (eg, cashiers, servers, restaurant workers, grocery store workers). Although we sampled throughout the United States, compared to the US census, the majority of the overall sample was European American (1538/1987, 77.4%, compared to the US census figure of 60%), with a somewhat higher representation of Asian Americans (238/1987, 12.0% vs 5% US census) and a lower representation of African Americans (172/1987, 8.7% vs 13% US census) and Latinx Americans (212/1956, 10.8% vs 18% US census) [45]. The sample was almost split evenly between male and female (female: 1027/1987, 52.2%).

Compared to the essential workers, the unemployed group had significantly more people who identified as being: Hispanic or Latinx, or an unlisted race; younger; any gender other than male; any sexuality other than straight; and never married. The group comprised significantly less White individuals. Of note, there were almost twice as many in the “single or never married” category than what would be expected compared to the US census data [46]; however, our sample was relatively young (ie, early 30s) compared to the US population [47]. Additionally, there were socioeconomic differences across groups. Compared to the essential workers, the unemployed group had significantly more individuals with lower education, less income, and lived somewhere other than a house or apartment.

Compared to participants that did not use a DMHT to cope with COVID-19 stress, DMHT users had a significantly higher proportion of individuals who identified as transgender and a lower proportion of individuals who identified as women or men. DMHT users were more likely to be married compared to non–DMHT users. In terms of socioeconomic differences, DMHT users had a significantly smaller proportion of individuals with lower levels of education and a higher percentage of individuals with higher education compared to non–DMHT users. Finally, compared to non–DMHT users, DMHT users were less likely to live in a house and more likely to live in an apartment.

**Table 1:** Sample characteristics.

Characteristic		Unemployed (n=1013)	Essential worker (n=974)		*P* value			Non–DMHT user (n=1680)			DMHT user (n=277)			*P* value			Total (N=1987)		
**Race (not mutually exclusive), n (%)**																			
	Asian American	129 (12.7)	109 (11.2)		.29^a^			207 (12.3)			29 (10.5)			.38^a^			238 (12.0)		
	European American/White	763 (75.3)	775 (79.6)		.02^a^			1300 (77.4)			225 (81.2)			.15^a^			1538 (77.4)		
	African American/Black	95 (9.4)	77 (7.9)		.24^a^			153 (9.1)			16 (5.8)			.07^a^			172 (8.7)		
	Hawaiian/Pacific Islander	8 (0.8)	3 (0.3)		.23^b^			11 (0.7)			0 (0)			.38^b^			11 (0.6)		
	American Indian/Alaska Native	24 (2.4)	24 (2.5)		.89^a^			36 (2.1)			9 (3.2)			.26^a^			48 (2.4)		
	Unlisted	52 (5.1)	22 (2.3)		<.001^a^			63 (3.8)			11 (4.0)			.86^a^			74 (3.7)		
**Ethnicity, (%)**						.003^a^									.91^a^				
	Hispanic/Latinx	128 (12.9)	84 (8.7)					179 (10.8)			30 (11.0)						212 (10.8)		
	Not Hispanic/Latinx	863 (87.1)	881 (91.3)					1483 (89.2)			243 (89.0)						1744 (89.2)		
**Age (years)**					<.001^c^									.92^c^					
	Mean (SD)	30.4 (11.1)	33.3 (9.9)					31.8 (10.8)			31.9 (9.7)						31.9 (10.6)		
	Range	18.0-73.0	18.0-78.0					18.0-78.0			18.0-73.0						18.0-78.0		
**Gender, n (%)**					<.001^b^									<.001^b^					
	Women	573 (57.4)	454 (46.9)					853 (51.1)			166 (60.1)						1027 (52.2)		
	Men	384 (38.4)	499 (51.5)					774 (46.3)			96 (34.8)						883 (44.9)		
	Nonbinary	35 (3.5)	12 (1.2)					38 (2.3)			9 (3.3)						47 (2.4)		
	Transgender	3 (0.3)	2 (0.2)					2 (0.1)			3 (1.1)						5 (0.3)		
	Unlisted	4 (0.4)	1 (0.1)					3 (0.2)			2 (0.7)						5 (0.3)		
**Sexuality, n (%)**					<.001^a^									.30^a^					
	Heterosexual/straight	681 (69.0)	802 (82.9)					1276 (76.6)			192 (71.6)						1483 (75.9)		
	Gay/lesbian/homosexual	69 (7.0)	41 (4.2)					89 (5.3)			20 (7.5)						110 (5.6)		
	Bisexual	189 (19.1)	104 (10.8)					243 (14.6)			45 (16.8)						293 (15.0)		
	Unlisted	48 (4.9)	20 (2.1)					57 (3.4)			11 (4.1)						68 (3.5)		
**Marital status, n (%)**					<.001^a^									.02^a^					
	Never married	737 (73.8)	500 (51.9)					1065 (63.8)			156 (57.6)						1237 (63.0)		
	Widowed	8 (0.8)	5 (0.5)					13 (0.8)			0 (0)						13 (0.7)		
	Married	177 (17.7)	402 (41.7)					473 (28.3)			101 (37.3)						579 (29.5)		
	Separated	15 (1.5)	7 (0.7)					21 (1.3)			1 (0.4)						22 (1.1)		
	Divorced	61 (6.1)	50 (5.2)					98 (5.9)			13 (4.8)						111 (5.7)		
**Education, n (%)**					<.001^a^									<.001^a^					
	High school graduate (or equivalent) or less	154 (15.3)	77 (7.9)					215 (12.8)			11 (4.0)						231 (11.7)		
	Some college	367 (36.5)	192 (19.7)					480 (28.6)			74 (26.7)						559 (28.3)		
	Trade/technical/vocational training/associate degree	125 (12.4)	108 (11.1)					209 (12.4)			22 (7.9)						233 (11.8)		
	Bachelor’s degree	283 (28.2)	353 (36.3)					540 (32.1)			92 (33.2)						636 (32.2)		
	Higher education (master’s, professional, or doctorate degree)	76 (7.6)	243 (25.0)					236 (14.0)			78 (28.2)						319 (16.1)		
**Income ($US), n (%)**					<.001^a^									.28^a^					
	<$10K	245 (25.2)	42 (4.4)					247 (15.0)			37 (13.7)						287 (14.8)		
	$10,000-$31,199	305 (31.3)	180 (18.7)					421 (25.6)			58 (21.4)						485 (25.1)		
	$31,200-$33,280	62 (6.4)	37 (3.9)					87 (5.3)			10 (3.7)						99 (5.1)		
	$33,281-$49,999	134 (13.8)	169 (17.6)					257 (15.6)			43 (15.9)						303 (15.7)		
	$50,000-$59,999	58 (6.0)	93 (9.7)					127 (7.7)			23 (8.5)						151 (7.8)		
	$60,000-$69,999	46 (4.7)	74 (7.7)					104 (6.3)			16 (5.9)						120 (6.2)		
	$70,000-$99,999	73 (7.5)	165 (17.2)					200 (12.2)			36 (13.3)						238 (12.3)		
	$100,000-$149,999	38 (3.9)	147 (15.3)					144 (8.8)			37 (13.7)						185 (9.6)		
	≥$150,000	13 (1.3)	54 (5.6)					56 (3.4)			11 (4.1)						67 (3.5)		
**Living situation, n (%)**					<.001^a^									.004^a^					
	House	611 (61.2)	624 (64.4)					1071 (64.0)			150 (54.7)						1235 (62.8)		
	Apartment	347 (34.7)	335 (34.6)					557 (33.3)			119 (43.4)						682 (34.7)		
	Other	41 (4.1)	10 (1.0)					45 (2.7)			5 (1.8)						51 (2.6)		

^a^Chi-square test.

^b^Fisher exact test.

^c^Unequal variance two-sample *t* test.

### Aim 1: Document Psychological Distress Among the Sample

Table 2 reports psychological distress (see the *Measures* section for calculation of the composite score) for the whole sample with stratification by employment status and DMHT-use status. We found that almost three-quarters of the sample fell into the “distressed” category (1479/1976, 74.8%), meaning they had scores at or above the clinical cut-off for at least one of the following: depression (PHQ-2), anxiety (GAD-2), risk for substance use disorder (CAGE-AID), risk for suicidal behaviors (SBQ-R), and history of suicide attempt. The unemployed group was more likely to be distressed than the essential worker group (815/1013, 81.2% vs 664/974, 68.3%; *χ*^2^_1_=43.40, *P*<.001; Table 2). DMHT users were significantly more likely to be distressed compared to non–DMHT users (236/277, 85.2% vs 1234/1680, 73.5%; *χ*^2^_1_=17.55, *P*<.001; Table 2). Table S1 in Multimedia Appendix 3 provides a further breakdown of depression, anxiety, risk for substance use disorder, risk for suicidal behaviors, and history of suicide attempt by total sample, employment status, and DMHT-use status.

**Table 2:** Psychological distress stratified by employment status and digital mental health tool (DMHT) use.

Variable			Unemployed (n=1013)		Essential worker (n=974)		*P* value		Non–DMHT user (n=1680)		DMHT user (n=277)		*P* value		Total (N=1987)
**Psychological distress, n (%)**							<.001^a,b^						<.001^a,b^		
	Nondistressed	189 (18.8)		308 (31.7)				446 (26.5)		41 (14.8)				497 (25.2)	
	Distressed	815 (81.2)		664 (68.3)				1234 (73.5)		236 (85.2)				1479 (74.8)	

^a^Chi-square test.

^b^*P* values <.05 and less than the Benjamini-Hochberg critical value were considered to be statistically significant.

### Aim 2: Explore DMHT Use in Response to COVID-19

Of the 1957 participants who responded, 277 (14.2%) reported using a DMHT to cope with stress associated with COVID-19. There was no significant difference in the proportion of participants who used a DMHT in the unemployed (137/1013, 13.5%) and essential worker (140/974, 14.4%) groups (*χ*^2^_1_=0.25, *P*=.62). Distressed individuals (236/1470, 16.1%) were significantly more likely to use a DMHT app compared to nondistressed individuals (41/487, 8.4%; *χ*^2^_1_=17.55, *P*<.001).

#### Most Used DMHTs

##### Total Sample

Among the total sample, which included 261 responses, the most used DMHTs were 2 meditation apps, Calm (41/261, 15.7%) and Headspace (38/261, 14.6%), followed by BetterHelp (11/261, 4.2%). A total of 119 participants (45.6%) reported using meditation apps, 25 (9.6%) reported using virtual therapy or DMHTs that facilitated contact with a virtual provider, and 21 (8.1%) used DMHTs with a chat feature (Table 3).

**Table 3:** Categories of digital mental health tools (DMHTs).

Category	Definition	Participants, n (%)^a^
Meditation/mindfulness	A DMHT offering primarily meditation or mindfulness (eg, Calm, Headspace)	119 (45.6)
Virtual therapy or contact with a virtual provider	A DMHT offering primarily virtual therapy via text, phone, or video, or appointments with a physician (eg, BetterHelp, Sanvello)	25 (9.6)
Chat feature	The main feature was a chat function for one-on-one chats with a peer or chatbot, group chats, or connecting with others in an organized forum (eg, Woebot, Wysa)	21 (8.1)
Health	Tools that offer education or tips to promote healthy habits with exercise, nutrition, physical health, or sleep (eg, Downdog)	20 (7.7)
COVID-19 contact tracing	A DMHT with information related to local COVID-19 cases, rates of infection, and information about symptoms or testing (eg, Contact Tracing)	13 (5.0)
Entertainment and distraction	A DMHT with entertainment, which may include movies, music, games, GIFs, memes, or other forms of entertainment (eg, Among Us, Music app)	12 (4.6)
Social media	A social media platform (eg, TikTok, Reddit)	10 (3.8)
Symptom tracking	A DMHT that allows users to monitor symptoms or daily activities (eg, eMoods, The Pattern)	10 (3.8)
COVID-19 coping	A DMHT providing emotional coping skills and education in the context of COVID-19 stressors (eg, COVID Coach)	8 (3.1)
Positive psychology	A DMHT with gratitude exercises or methods to promote positivity, such as daily verses, positive thoughts, uplifting stories, or uplifting quotes (eg, InnerHour)	7 (2.7)
Finance	A DMHT with resources for financial decisions, financial decision-making, or spending tips (eg, Yes, Pacific)	7 (2.7)
Journal	A DMHT with primarily writing or journaling features (eg, Day One, Iona)	4 (1.5)
News	Information about international or national occurrences (eg, WHO Info)	3 (1.2)
Crisis	Using a DMHT to manage crisis or safety (eg, suicide)	1 (0.4)
Language learning	Using a DMHT in order to practice or learn a new language	1 (0.4)

^a^A total of 18 responses were coded as “missing” due to being indecipherable or unidentifiable; percentages do not reflect missingness.

##### Employment Status

The leading entries by the unemployed sample were 3 meditation apps: Calm (26/131, 19.8%), Headspace (22/131, 16.8%), and Insight Timer (7/131, 5.3%). The most common DMHT categories among individuals unemployed due to COVID-19 were meditation (70/131, 53.4%), virtual therapy or DMHTs that facilitated virtual contact with a mental health provider (11/131, 8.4%), and DMHTs with a chatbot (11/131, 8.4%). The most frequently reported DMHTs by the essential worker sample were Headspace (16/130, 12.3%), Calm (15/130, 11.5%), and COVID Coach (8/130, 6.2%). By category, essential workers reported using mostly meditation (49/130, 37.7%), DMHTs with virtual therapy or contact with a virtual provider (14/130, 10.8%), health DMHTs (12/130, 9.4%), and COVID-19 contact tracing (12/130, 9.4%).

##### Distress Status

Similarly, the leading entries by the distressed sample were 2 meditation apps, Calm (33/223, 14.8%) and Headspace (32/223, 14.3%), followed by BetterHelp (10/223, 4.5%). Most of the distressed sample used meditation (100/223, 44.8%), virtual therapy or contact with a virtual provider (24/223, 10.8%), and DMHTs with a chat feature (19/223, 8.5%). The most frequently reported DMHTs by the nondistressed group were Calm, (8/38, 21.1%), Headspace (6/38, 15.8%), and COVID Coach (2/38 5.3%). Among the individuals in the nondistressed group, the most frequently used app categories were meditation (9/38, 50%), COVID-19 contact tracing (4/38, 10.5%), and social media (3/38, 7.9%).

Further comparisons of app categories by employment and distress statuses may be found in Table S2 (Multimedia Appendix 3).

### Reasons for Lack of Use

Most of the sample (1710/1957, 85.9%) reported that they did not use a DMHT to cope with COVID-19. The primary reasons for not using a DMHT to cope with COVID-19 were (1) not thinking to look for an app (1179/1710, 68.9%), (2) not thinking apps would help them (605/1710, 35.4%), and (3) having other ways of coping (421/1710, 24.6%). Table S3 in Multimedia Appendix 3 lists all reasons for lack of use. These top 3 responses were endorsed by all subgroups.

There were differences that emerged by employment status and distress status. Compared to essential workers, those who were unemployed due to COVID-19 were more likely to report not thinking to look for a DMHT (629/876, 71.8% vs 550/834, 65.9%; *χ*^2^_1_=6.84, *P*=.009) and not having money to spend on a data plan to use a DMHT (112/876, 12.8% vs 54/834, 6.5%; *χ*^2^_1_=19.41, *P*<.001).

Compared to the nondistressed group, distressed individuals were more likely to not think to look for an app (293/456, 64.3% vs 886/1243, 71.3%; *χ*^2^_1_=7.75, *P*=.005), to not think apps would help them (142/456, 31.1% vs 463/1243, 37.2%; *χ*^2^_1_=5.43, *P*=.02), to prefer working with a professional (32/456, 7.0% vs 191/1243, 15.4%; *χ*^2^_1_=20.39, *P*<.001), to not have money to spend on a data plan to use apps (25/456, 5.5% vs 141/1243, 11.3%; *χ*^2^_1_=13.00, *P*<.001), and to not find an app that was relevant to their needs (19/456, 4.2% vs 103/1243 8.3%; *χ*^2^_1_=8.50, *P*=.004). However, compared to nondistressed individuals, distressed workers were less likely to state that having another way of coping was the reason for why they did not use a DMHT (281/1243, 22.6% vs 140/456, 30.7%; *χ*^2^_1_=11.73, *P*<.001).

### Aim 3: Assess DMHT Usability and User Burden

Data for the following analyses were taken from the 277 participants who reported using a DMHT to cope with COVID-19. Individuals who did not report using a DMHT to cope with COVID-19 did not complete the SUS or UBS (Figure 1).

#### Employment Status

As shown in Table 4, compared to the essential workers, those who were unemployed due to COVID-19 reported significantly less user burden when using DMHTs (mean 13.69, SD 17.76 vs mean 7.23, SD 8.24; *t*_198.1_=–3.89, *P*<.001). Specifically, those who were unemployed rated their selected DMHT as being significantly less difficult to use (mean 2.77, SD 4.02 vs mean 1.53, SD 2.15; *t*_214.5_=3.20, *P*=.002), and having less physical burden (mean 1.54, SD 2.89 vs mean 0.43, SD 1.37; *t*_198.3_=4.06, *P*<.001), time and social burden (mean 2.60, SD 4.00 vs mean 1.07, SD 2.15; *t*_215.1_=3.95, *P*<.001), mental and emotional burden (mean 2.46, SD 3.92 vs mean 1.07, SD 2.08; *t*_213.4_=3.69, *P*<.001), and privacy burden (mean 2.30, SD 3.16 vs mean 1.25, SD 2.14; *t*_245.1_=3.25, *P*=.001). The conditions did not differ for reports of financial burden (mean 2.04, SD 2.33 vs mean 1.88, SD 2.47; *t*_272_=–0.53 *P*=.59). In addition, there was no significant difference in ratings of usability between unemployed individuals (mean 76.96, SD 16.21) and essential workers (mean 74.32, SD 17.01; *t*_271_=–1.31, *P*=.19).

**Table 4:** User burden and system usability stratified by workers and psychological distress.

Variable		Unemployed (n=137)	Essential worker (n=140)	*P* value	Nondistressed (n=41)	Distressed (n=236)	*P* value	Total (N=277)
**Overall burden**				<.001^b,c^			.30^a^	
	Count, n	134	140		41	233		274
	Mean (SD)	7.2 (8.2)	13.7 (17.8)		8.4 (13.8)	10.9 (14.4)		10.5 (14.3)
**Difficulty of use**				.002^b,c^			.42^a^	
	Count, n	134	140		41	233		274
	Mean (SD)	1.5 (2.2)	2.8 (4.0)		1.8 (3.8)	2.2 (3.2)		2.2 (3.3)
**Physical burden**				<.001^b,c^			.52^a^	
	Count, n	134	139		40	233		273
	Mean (SD)	0.4 (1.4)	1.5 (2.9)		0.8 (2.0)	1.0 (2.4)		1.0 (2.3)
**Social and time burden**				<.001^b,c^			.99^a^	
	Count, n	134	140		41	233		274
	Mean (SD)	1.1 (2.2)	2.6 (4.0)		1.9 (3.3)	1.9 (3.3)		1.9 (3.3)
**Mental and emotional burden**				<.001^b,c^			.80^a^	
	Count, n	134	140		41	233		274
	Mean (SD)	1.1 (2.1)	2.5 (3.9)		1.7 (3.3)	1.8 (3.2)		1.8 (3.2)
**Privacy burden**				.001^b,c^			.22^a^	
	Count, n	134	140		41	233		274
	Mean (SD)	1.2 (2.1)	2.3 (3.2)		1.3 (2.6)	1.9 (2.8)		1.8 (2.8)
**Financial burden**				.59^a^			.002^b,c^	
	Count, n	134	140		41	233		274
	Mean (SD)	1.9 (2.5)	2.0 (2.3)		1.1 (1.8)	2.1 (2.5)		2.0 (2.4)
**System Usability Score**				.19^a^			.96^a^	
	Count, n	134	139		40	233		273
	Mean (SD)	77.0 (16.2)	74.3 (17.0)		75.5 (17.6)	75.6 (16.5)		75.6 (16.6)

^a^Equal variance two-sample *t* test.

^b^Unequal variance two-sample *t* test.

^c^*P* values <.05 and less than the Benjamini-Hochberg critical value were considered to be statistically significant.

#### Distress Status

As shown in Table 4, there was no difference in reported DMHT burden between the distressed and nondistressed subsamples (mean 10.91, SD 14.37 vs mean 8.41, SD 13.82; *t*_272_=–1.03, *P*=.30) or in overall usability (mean 75.63, SD 16.52 vs mean 75.50, SD 17.59; *t*_271_=–0.05, *P*=.96). Likewise, we found no difference between groups in types of burden (Table 4). The one exception was that distressed individuals reported higher financial burden for their selected DMHT than nondistressed individuals (mean 2.12, SD 2.46 vs mean 1.07, SD 1.81; *t*_69.0_=–3.21, *P*=.01).

Finally, we explored the user burden and usability ratings of the three most used apps (ie, Calm, Headspace, and BetterHelp; shown in Table S4 in Multimedia Appendix 3). There were no statistically significant differences among the apps in terms of the total SUS, total UBS, and UBS subscales, except for privacy burden (Calm: mean 1.54, SD 2.82 vs Headspace: mean 0.50, SD 1.03 vs BetterHelp: mean 2.00, SD 2.14; *F*_2.87_=3.25, *P*=.04).

### Aim 4: Identify Important DMHT Features

#### Total Sample

The sample reported the following top-rated features for DMHTs: (1) information or education (mean 6.09, SD 2.66); (2) mindfulness or meditation tools (mean 6.06, SD 2.59); (3) link to resources, counseling, or crisis support (mean 5.93, SD 2.80); and (4) tools to focus on positive events and influences in life (mean 5.88, SD 2.46).

Participants also had the option to write in what DMHT features they felt were important to include but were not provided in the list of options. The top suggested features among the 764 responses were the ability to chat with a mental health professional, support personnel, or peer (n=57); entertainment and distraction (n=39); and positive psychology (n=29). The feature “entertainment and distraction” was defined as “different forms of entertainment such as music, movies, movie clips, GIFs, memes, games, or other forms of distraction.” Additionally, participants reported wanting regularly occurring (ie, daily) gratitude exercises or activities to promote positivity, such as verses, quotes, and uplifting or hopeful stories, which we categorized as “positive psychology” features. Example responses included: “give positive messages in the morning or something like that,” “daily gratitude,” and “a good news section… I don’t want to be told COVID-19 isn’t a problem. I want to know what hope there is.”

When provided the option to build their own app, the sample most frequently endorsed the following features: mindfulness/meditation (1271/1987, 64.0%), information or education (1254/1987, 63.1%), and distraction tools (1170/1987, 58.9%) (Table 5).

**Table 5:** Digital mental health tool (DMHT) features stratified by worker status and psychological distress.

Feature	Unemployed (n=1013), n (%)	Essential worker (n=974), n (%)	*P* value	Nondistressed (n=497), n (%)	Distressed (n=1479), n (%)	*P* value
Mindfulness/meditation	687 (67.8)	584 (60.0)	<.001^a,b^	305 (61.4)	966 (65.3)	.11^a^
Information or education	636 (62.8)	618 (63.4)	.76^a^	327 (65.8)	927 (62.7)	.21^a^
Distraction tools (drawing, puzzles, music)	630 (62.2)	540 (55.4)	.002^a,b^	276 (55.5)	894 (60.4)	.05^a^
Symptom tracking (tracking sleep or mood)	605 (59.7)	555 (57.0)	.22^a^	270 (54.3)	890 (60.2)	.02^a,b^
Link to resources, counseling, or crisis support	604 (59.6)	536 (55.0)	.04^a,b^	276 (55.5)	864 (58.4)	.26^a^
Tools to focus on the positive events and influences in life	578 (57.1)	553 (56.8)	.90^a^	267 (53.7)	864 (58.4)	.07^a^
Brain games to improve thinking	525 (51.8)	480 (49.3)	.26^a^	257 (51.7)	748 (50.6)	.66^a^
How to cope with COVID-19	406 (40.1)	409 (42.0)	.39^a^	200 (40.2)	615 (41.6)	.60^a^
A chatbot to help you with daily stress	352 (34.7)	293 (30.1)	.03^a,b^	139 (28.0)	506 (34.2)	.01^a,b^

^a^Chi-square test.

^b^*P* values <.05 and less than the Benjamini-Hochberg critical value were considered to be statistically significant.

#### Employment Status

The three most important DMHT components for essential workers and unemployed individuals were information or education (essential: mean 6.09, SD 2.70; unemployed: mean 6.09, SD 2.61); mindfulness/meditation (essential: mean 6.17, SD 2.55; unemployed: mean 5.94, SD 2.62); and link to resources, counseling, or crisis support (essential: 6.00, SD 2.89; unemployed: mean 5.86, SD 2.72). Unemployed participants were more likely to rate distraction tools (drawing, puzzles, and music) (mean 5.84, SD 2.55 vs mean 5.42, SD 2.59; *t*_1945_=3.59, *P*<.001) and mindfulness/meditation (mean 6.17, SD 2.55 vs mean 5.94, SD 2.61; *t*_1945_=2.02, *P*=.04) as more important than essential workers.

When provided the option to build their own DMHT, the most common features listed by essential workers were information and education (618/974, 63.4%), mindfulness/meditation (584/974, 60.0%), and symptom tracking (tracking sleep or mood; 555/974, 57%). The most common features reported by unemployed persons was mindfulness/meditation (687/991, 67.8%), information or education (636/991, 62.8%), and distraction tools (eg, drawing, puzzles, music) (630/991, 62.2%). In comparing the desired features for a DMHT by employment status, unemployed participants were more likely to request that their DMHT include mindfulness/meditation (687/1013, 67.8% vs 584/974, 60.0%; *χ*^2^_1_=13.31, *P*<.001); distraction tools (drawing, puzzles, and music; 630/1013, 62.2% vs 540/974, 55.4%; *χ*^2^_1_=9.34, *P*=.002); link to resources, counseling, or crisis support (604/1013, 59.6%, vs 536/974, 55.0%; *χ*^2^_1_=4.29, *P*=.04); and a chatbot to help with daily stress (352/1013, 34.7%, vs 293/974, 30.1%; *χ*^2^_1_=4.93, *P*=.03) than the essential worker group (Table 5).

#### Distress Status

The most important DMHT components among distressed and nondistressed users included information or education (distressed: mean 6.01, SD 2.67; nondistressed: mean 6.32, SD 2.59); mindfulness/meditation (distressed: mean 6.09, SD 2.56; nondistressed: mean 5.96, SD 2.68); and link to resources, counseling, or crisis support (distressed: mean 5.95, SD 2.81; nondistressed: mean 5.88, SD 2.80). Distressed individuals also rated tools to focus on positive life events and influences as important (mean 5.90, SD 2.42).

When provided the option to build their own DMHT, nondistressed individuals indicated information or education (327/497, 65.8%), followed by mindfulness/meditation (305/497, 61.4%), distraction tools (276/497, 55.5%), and link to resources, counseling, or crisis support (276/497, 55.5%). Similarly, distressed individuals desired to include mindfulness/meditation (966/1479, 65.3%), followed by information or education (927/1479, 62.7%) and distraction tools (894/1479, 60.4%). Compared to nondistressed individuals, distressed participants preferred to include symptom tracking (270/497, 54.3% vs 890/1479, 60.2%; *χ*^2^_1_=5.25, *P*=.02) and a chatbot (139/497, 28.0% vs 506/1479, 34.2%; *χ*^2^_1_=6.60, *P*=.01) within their DMHT (Table 5).

#### DMHT Use Status

Participants who used DMHTs to cope during COVID-19 reported the following features as having the highest importance for a DMHT: (1) mindfulness/meditation (mean 7.10, SD 2.05); (2) tools to focus on the positive events and influences in life (mean 6.23, SD 2.24); (3) link to resources, counseling, or crisis support (mean 5.94, SD 2.64); and (4) symptom tracking (mean 5.90, SD 2.40). On the other hand, non–DMHT users indicated their most important features were (1) information or education (mean 6.14, SD 2.69); (2) link to resources, counseling, or crisis support (mean 5.93, SD 2.83); and (3) mindfulness/meditation tools (mean 5.89, SD 2.63).

When asked to build their own DMHT, individuals who did not use a DMHT to cope during the COVID-19 pandemic preferred to include information or education (1091/1680, 64.9%), mindfulness/meditation (1071/1680, 63.8%), and distraction tools (1031/1680, 61.4%). DMHT users preferred to include mindfulness/meditation (200/277, 72.2%), tools to focus on the positive events and influences in life (178/277, 64.3%), and symptom tracking (tracking sleep or mood; 166/277, 59.9%).

Participants who used DMHTs to cope during COVID-19 were more likely than those who did not use DMHTs to prefer mindfulness/meditation features (200/277, 72.2% vs 1071/1680, 63.8%; *χ*^2^_1_=7.46, *P*=.006), positive psychology features (178/277, 64.3% vs 953/1680, 56.7%; *χ*^2^_1_=5.53, *P*=.02), and chatbot features (108/277, 39.0% vs 537/1680, 32.0%; *χ*^2^_1_=5.31, *P*=.02). Conversely, compared to non–DMHT users, DMHT users were less likely to prefer brain games to improve thinking (124/277, 44.8% vs 881/1680, 52.4%; *χ*^2^_1_=5.61, *P*=.02), and distraction tools (139/277, 50.2% vs 1031/1680, 61.4%; *χ*^2^_1_=12.38, *P*<.001) (Table 6).

**Table 6:** Digital mental health tool (DMHT) features stratified by user status.

Feature	Non–DMHT user (n=1680), n (%)	DMHT user (n=277) , n (%)	*P* value	Total (N=1987), n (%)
Mindfulness/meditation	1071 (63.8)	200 (72.2)	.006^a,b^	1271 (64.0)
Information or education	1091 (64.9)	163 (58.8)	.05^a^	1254 (63.1)
Distraction tools (drawing, puzzles, music)	1031 (61.4)	139 (50.2)	<.001^a,b^	1170 (58.9)
Symptom tracking (tracking sleep or mood)	994 (59.2)	166 (59.9)	.81^a^	1160 (58.4)
Link to resources, counseling, or crisis support	986 (58.7)	154 (55.6)	.33^a^	1140 (57.4)
Tools to focus on the positive events and influences in life	953 (56.7)	178 (64.3)	.02^a,b^	1131 (56.9)
Brain games to improve thinking	881 (52.4)	124 (44.8)	.02^a,b^	1005 (50.6)
How to cope with COVID-19	689 (41.0)	126 (45.5)	.16^a^	815 (41.0)
A chatbot to help you with daily stress	537 (32.0)	108 (39.0)	.02^a,b^	645 (32.5)

^a^Chi-square test.

^b^*P* values <.05 and less than the Benjamini-Hochberg critical value were considered to be statistically significant.

## Discussion

### Principal Findings

This study documented DMHT use among essential workers and unemployed individuals during the COVID-19 pandemic and determined which features such users would prefer to have in a DMHT offering. DMHT use has been deemed by many in the field to be subpar, and some have suggested that poor uptake and adherence to such tools is the result of user burden and inadequate match to user needs [17]. Indeed, our findings indicate that despite reports of increased downloads [48] and user registration by digital mental health companies [13], use of DMHTs by essential workers and those unemployed due to COVID-19 is very similar to prepandemic reports (14%). Compared to our study (14%), previous studies found that 10% of outpatient psychiatric clinic patients used a DMHT [49] and only 17% of a sample with no self-reported mental health distress report downloading an app “to help relax” [50].

Of those who reported using a DMHT, by far the most common DMHTs were those that focused on mindfulness/meditation strategies (46%), with access to virtual therapy (10%) in second place. This finding did not vary by level of distress or employment status except among the nondistressed group using COVID-19 contact tracing (8% of this subsample). This finding is nearly identical to another recent study that found that Calm and Headspace were the top 2 downloaded apps among iPhone users during COVID-19 [48].

Additionally, when asked to rate the usability and user burden of the DMHT tool participants used the most, system usability fell in the “acceptable” range [34], and time, mental and emotional, physical, financial, and privacy burdens were seen as acceptable, with essential workers finding these tools to be more burdensome than the unemployed group. Increased perceived burdensome may be partially explained by previous findings suggesting that essential workers have increased fatigue from elevated anxiety and work demands during the ongoing pandemic [3].

Individuals with increased mental health needs (ie, the distressed group) reported more financial burden of DMHTs than the nondistressed. It is understandable that during a pandemic, where people are struggling financially, there would be concerns about the costs of DMHTs, given that many popular and widely publicized tools require a paid subscription. In the United States, those who lost their jobs during COVID-19 are faced with insufficient insurance to cover the costs of mental health care [51], and those who are struggling financially likely have additional financial concerns aside from a DMHT subscription fee, such as the cost of data plans and the technology needed to use these services. In fact, an earlier study noted that most individuals with depression and/or anxiety symptoms preferred using health apps that were free or had low cost for download (eg, <$5) [43]. As such, reimbursement is one part of the solution for increasing access to care for everyone, and until technology is more affordably available to all, the use of these services will be compromised [52].

**Figure 2:**
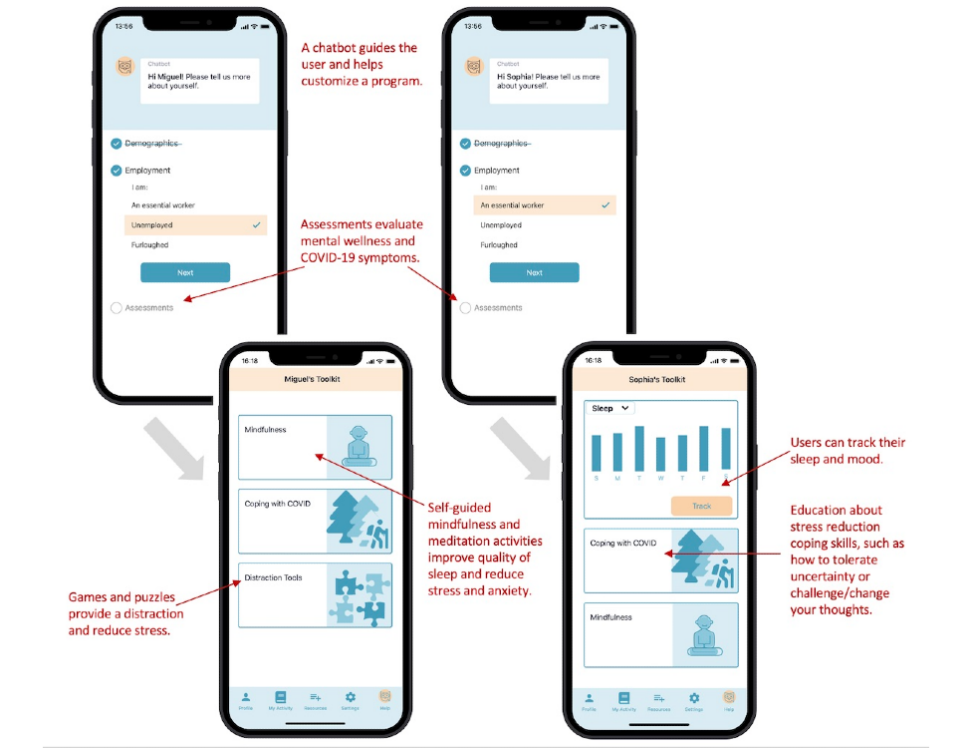
Preferred digital mental health tool features according to participants.

When asked to design their own DMHT for coping with COVID-19, again mindfulness/meditation was listed as an important feature for all subgroups in this study. Interestingly, information and education about COVID-19 was also consistently listed as an important feature in all subgroups except for people who had used DMHTs during the pandemic. In addition to mindfulness/meditation, people who used DMHTs to cope with COVID-19 preferred positive psychology tools and mood and sleep tracking. Figure 2 illustrates the preferences between the unemployed and essential worker groups. This finding has important implications for DMHT development focused on pandemic response and other prolonged environmental disasters. Developers would be able to create a single tool that includes mindfulness/meditation, information and education about COVID-19 coping, and distraction tools, which would appeal to a wide group of people with different needs during COVID-19, with only a few added features for specific populations.

A final finding in this study was reasons for not turning to DMHTs to cope with COVID-19. Most of the sample indicated that they did not use a DMHT because they did not think to look for such a tool. Past reports suggest that this result may be due to a lack of information about how DMHTs might be effective [53]. This assumption is further supported by the fact that one-third of the sample did not think a DMHT would be helpful to them, and one-quarter of the sample indicated that they had other means of coping. The potential lack of confidence in DMHTs might be addressed through education to health providers on the effectiveness of DMHTs [54], the creation of reimbursement codes in the United States that would allow providers to prescribe these services [55], or the further use of a human-centered design from DMHT companies to create tools that are appropriately targeting user needs and concerns.

### Comparison With Prior Work

A strength of this study is that we explicitly asked a large sample of users about their app preferences and perceived importance of various features. This survey was different from previous studies that have primarily focused on downloads and user metrics [48], insight from providers and private digital health companies [56], and self-report from individuals exclusively with mild depression or mild anxiety symptoms with exclusion of severer mental health conditions (eg, suicidality) [43]. It is also novel in its consideration of user-centered design principles (eg, ease of use and learnability) when developing and identifying DMHT features that would be most acceptable to a very large sample of potential target consumers. Consistent with emerging models that integrate community-based research, implementation science, and user-centered design principles [57,58], this is an important first step in a well-planned process of DMHT design to identify the needs and preferred features that users, both experienced and unexperienced, and preferences for what tools they would like to see in a DMHT. Previous studies that used self-report of physical health and mental health apps found that users typically only use an app for one feature [43]. It might be that future apps need to have multiple features incorporated to meet the overarching needs of similar populations. As Mohr et al [17] have noted previously, health app developers tend to create a tool based on what the developer feels is essential and historically only designs around these developer-driven features, rather than asking the end-user what role they see digital health playing in their lives, what needs they have that are unmet, and what functions they want these tools to have. By starting with understanding end-user needs and preferences, DMHT developers may see not only an increase in DMHT uptake but long-term use as well.

The findings of this study differ from findings in recent studies on the use of technology to cope with the consequences of COVID-19. According to recent research in the general population, there has been increased desire for apps or online resources that allow for fitness at home, owing to physical distancing and stay-at-home orders that have led to a shift from gyms and group fitness classes to exercise at home [59]. During prepandemic times, Rubanovich et al [43] found that people with depression and anxiety symptoms reported more frequently using health apps featuring fitness, pedometers, or heart rate monitoring apps than DMHTs. Conversely, in our study, fitness apps and tools were listed very low in the list of tools participants used for coping with COVID-19. Although studies on the use of fitness apps among essential workers and employee groups are sparse, existing research suggests that the use of such tools in practice is low [60], which may explain why these tools were not in the top group of DMHTs listed by these participants. According to past research, those who are unemployed may likewise not have resources to engage in fitness apps, and generally are less likely to engage in fitness tracking [61]. Finally, another COVID-19 study found that more contact tracing and COVID-19 informational apps were being downloaded than DMHTs in North America [62]. We note here that downloads are often not equivalent to tool use as recent research has found that many people do download such tools but rarely use them long term [20,63]. Our study specifically asked about which DMHTs people used to cope with COVID-19 stress.

Our study adds to the existing body of work by understanding how DMHTs could be made to be more accessible to those at risk for the emotional consequences of COVID-19. Many experts in digital mental health have argued for the need to better personalize such tools [54] and to include the perspectives of the intended consumer in the design of such tools [8].

### Limitations

Although this study has important implications regarding the use of DMHTs from a human-centered design approach, it does have limitations. First, this is a cross-sectional study surveying the US participants’ experiences and opinions at one point in time. Second, the participants of this sample are likely to be more accepting of digital tools, as they were recruited from an online research platform. As such, the information from this study is limited to those who are currently using and familiar with technology. Third, this study did not consider cross-cultural acceptance of DMHTs, which is an important caveat since a DMHT may be different in countries that already support such tools as part of their health care system. Fourth, we are unable to explicitly comment on the sample’s overall experience with apps or DMHTs during prepandemic times. The focus of this paper was to explore whether users were using available, low-cost DMHTs to address COVID-19–related stress. Future studies should conduct a more thorough assessment of both current and previous DMHT use.

### Conclusions

Despite the limitations, this study provides important information to the mental health care system and to those who develop and provide DMHTs during prolonged stressful events. Policy makers and providers may not be able to rely on existing DMHTs to address the emotional health of essential workers and people who are unemployed. This study points to the need to ensure DMHTs address the needs that the intended consumer feels is most important, that these tools are not burdensome under high-stress conditions, and that they are affordable to people who have limited means.

## Appendix

Multimedia Appendix 1 Consent form and COVID-19 mental health apps survey.

Multimedia Appendix 2 Distress measures stratified by app users.

Multimedia Appendix 3 Additional supplementary tables.

## Abbreviations

ANOVA: analysis of variance
CAGE-AID: Cut-Annoyed-Guilty-Eye Adapted to Include Drugs
DMHT: digital mental health tool
GAD-2: 2-item Generalized Anxiety Disorder
MIND: M-Health Index and Navigation Database
PHQ-2: 2-item Patient Health Questionnaire
SBQ-R: Suicide Behaviors Questionnaire–Revised
UBS: Use Burden Scale
SARS: severe acute respiratory syndrome
SUS: System Usability Scale
VOIP: voice over IP
