# Supplementary material for: Mental Health and the Perceived Usability of Digital Mental Health Tools Among Essential Workers and People Unemployed Due to COVID-19: Cross-sectional Survey Study
Source: JMIR Ment Health. 2021 Aug 5;8(8):e28360. doi: 10.2196/28360 (PMC8354319; doi:10.2196/28360)
Supplement: Multimedia Appendix 1 [file mental_v8i8e28360_app1.doc]

**Consent Form**

**UNIVERSITY OF WASHINGTON**

**Acceptability, Usability and Effectiveness of Mental Health Apps for Suicide Prevention in**
**Essential Workers and the Unemployed during COVID-19**
**Survey Phase Consent Form**

The University of Washington is conducting a research study to learn about the acceptability, feasibility, usability, and effectiveness of mobile mental health apps for addressing risk factors associated with suicide risk in essential workers and unemployed individuals during COVID-19.

**STUDY PROCEDURES**
We will be asking you questions about your mental health needs, challenges that you have faced receiving care, and strategies that you have used to manage your mental health. We will ask you to review descriptions of mobile mental health applications and provide initial impressions on the acceptability and usability of these apps. We anticipate that answering these questions will take about 15 minutes. Some of the questions that we ask may be uncomfortable to answer since we are asking some personal questions about your mood and mental health, such as have you attempted to kill yourself. You may refuse to answer any question at any time and continue with the study. The interview may result in fatigue; however, you may take breaks during the interview if you choose.

Upon completion of the questionnaires, you will be paid $3. We will also ask you if you would like to receive information on other phases of this study.

**SOURCE OF FUNDING**
The University of Washington is receiving financial support from the National Institute of
Mental Health. A description of this clinical trial will be available on [http://www.clinicaltrials.gov](http://www.clinicaltrials.gov/), as required by U.S. Law. This Web site will not include information that can identify you. At most, the Web site will include a summary of the results. You can search this Web site at any time.

**BENEFITS OF THE STUDY**
There will be no direct benefit to you from participating in the study, although you will receive information on mobile mental health apps that you may find helpful.

**CONFIDENTIALITY OF RESEARCH INFORMATION**
The study team maintains the confidentiality of research information, meaning that no one other than the people working on the study will see your answers to the questions, unless required by law. We have a Certificate of Confidentiality from the federal National Institutes of Health. This helps us protect your privacy. The Certificate means that we do not have to give out information, documents, or samples that could identify you even if we are asked to by a court of law. We will use the Certificate to resist any demands for identifying information. We can't use the Certificate to withhold your research information if you give your written consent to give it to an insurer, employer, or other person. Also, you or a member of your family can share information about yourself or your part in this research if you wish. There are some limits to this protection. We will voluntarily provide the information to:
**• a member of the federal government who needs it in order to audit or evaluate the**
**research;**
• individuals at the institution(s) conducting the research, the funding agency, and
other groups involved in the research, if they need the information to make sure the
research is being done correctly;
• the federal Food and Drug Administration (FDA), if required by the FDA;
• individuals who want to conduct secondary research if allowed by federal
regulations and according to your consent for future research use as described in
this form;
• local authorities, if we learn of child abuse, elder abuse, or the intent to harm
yourself or others.
The Certificate expires when the NIH funding for this study ends. Currently this is April 30, 2022. Any data collected after expiration is not protected as described above. Data collected prior to expiration will continue to be protected.

**USE OF INFORMATION**
The information and/or data that we obtain from you for this study might be used for future research. We will have no access to anything that might identify you so nothing identifiable would be shared. If we do so, that information and data may then be used for future research studies or given to another investigator without getting additional permissions from you. It is also possible that in the future we may want to use or share study information that might identify you. If we do, an independent review board will decide whether or not we need to get additional permissions from you.

**OTHER INFORMATION**
Participation in research is voluntary and you may refuse or discontinue with no penalty. If you have questions about the research or think you have been harmed, you may contact the study team at (206) 744-1752 or [uwcspar@uw.edu](mailto:uwcspar@uw.edu). If you have questions about your rights as a subject, you can contact the UW Human Subjects Division at [hsdinfo@uw.edu](mailto:hsdinfo@uw.edu).

If you would like to download a copy of this consent form for your records, click on the PDF attached below:

*(Option to download PDF of consent)*

**Because this is a research study, it is important that you know what is going to happen. Please answer these questions:**

**TRUE/FALSE: Being in the research study is voluntary**

*If “TRUE,” then “That’s right! Being in this study is completely voluntary and you can stop at any time.”*

*If “FALSE,” then “Good try! Actually, being in this study is voluntary and you can stop at any time.”*

**TRUE/FALSE: No one other than the people working on the study will see my answers to the questions, unless the study is being reviewed by individuals at UW, the funding agency, and other groups involved in the research if they need the information to make sure the research is being done correctly.**

*If “TRUE,” then “That’s right! We will keep all of your answers private, unless the study is being reviewed by individuals at UW, the funding agency, and other groups involved in the research if they need the information to make sure the research is being done correctly.”*

*If “FALSE,” then “*Nice try! We actually do keep all of your answers private, unless the study is being reviewed by individuals at UW, the funding agency, and other groups involved in the research if they need the information to make sure the research is being done correctly.”

**TRUE/FALSE: Answering these questions will probably take about 15 minutes.**

*If “TRUE,” then “That's right! We think that this should take about 15 minutes.*

*If “FALSE,” then “Almost! We think that this should take about 15 minutes.”*

Thanks for answering our questions. Now is the time to let us know whether or not you would like to participate in the study. Please select whether or not you would like to participate.

Yes/No

*If “Yes,” By clicking "Submit" below, you are consenting to participate in the survey.*

*If “No,” then “Thank you. Please close the survey now.”*

**Inclusion Screening**

1. Are you currently employed and working (have a paid job and have not been laid off or furloughed due to the COVID-19 pandemic)?

Yes

No

*If “Yes,” to #1, then ask:*

2. What is your job (e.g.,) what type of work did you do? *(Open-ended)*

3. Are you considered an essential worker during the COVID-19 pandemic?

Yes

No

*If “No,” to #1, then ask:*

4. Have you become unemployed as a result of the COVID-19 pandemic?

Yes

No

*If “Yes” to #4, then ask:*

5. What job did you hold prior to becoming unemployed as a result of the COVID-19 pandemic? *(Open-ended)*

*If “No” to #3 and #4, then “Thank you for your interest in the study. You are not eligible to participate. Please exit the survey now.*

*If “Yes” to #3 or #4, then “You are eligible to participate in the study. Please click “Submit” to continue the survey.*

**Attention Check**

*(Embedded within the survey)*

To confirm that you are still paying attention, select “Strongly Disagree.”

Strongly Disagree

Disagree

Neutral

Agree

Strongly Agree

**Demographics**

What is your Prolific ID? *(open-ended)*

What city do you live in? *(open-ended)*

What state do you live in?

What is your zip code? *(open-ended)*

What is your race? (check all that apply)

Asian

White

African American/Black

Hawaiian/Pacific Islander

American Indian/Alaska Native *(open-ended for tribal affiliation)*

Other  *(open-ended)*

What is your ethnicity?

Hispanic/Latinx

Not Hispanic/Latinx

Choose not to answer

What is your age? *(open-ended)*

To what gender identity do you most identify? *(open-ended)*

Do you consider yourself to be:

Heterosexual/straight

Gay/lesbian/homosexual

Bisexual

Other *(open-ended)*

I cannot/do not want to answer

What is your current marital status?

Never married

Widowed

Married (including same sex partnership)

Separated

Divorced

I cannot/do not want to answer

What is your highest level of education?

No schooling completed

8^th^ grade

Some high school, no diploma

High school graduate or equivalent

Some college, no degree

Trade/technical/vocational training

Associate degree

Bachelor’s degree

Master’s degree

Professional or doctorate

Please estimate your gross annual income (before taxes) for the last year based on routine/typical income sources (such as your or partner's income, unemployment, or disability payments; don't include one-time sources of income or subsidies for housing, food, etc.)

Below $10K

$10,000-$31,199

$31,200-$33,280

$33,281-$49,999

$50,000-$59,999

$60,000-$69,999

$70,000-$99,999

$100,000-$149,999

$150,000 or above

I cannot/do not want to answer

What is your current living situation (apartment, house, clean and sober housing, shelter, etc.)?

House

Apartment

Clean and sober housing

Shelter

Couch surfing

Street homeless (car, street no heat or running water)

Other *(open-ended)*

I cannot/do not want to answer

**Mental Health and Possible Substance Use Disorder**

**PHQ-2**

This next set of questions is related to any low or depressed mood you may have been experiencing in last 2 weeks. Please respond using the following answer scale: 0 - Not at all sure, 1 - Several Days, 2 - More than half the days, or 3- Nearly Every day. Please indicate what feels most accurate for you when thinking about the last 2 weeks.

1. Little interest or pleasure in doing things

Not at all (0)

Several days (1)

More than half the days (2)

Nearly every day (3)

Choose not to answer

1. Feeling down, depressed, or hopeless

Not at all (0)

Several days (1)

More than half the days (2)

Nearly every day (3)

Choose not to answer

**GAD-2**

Now we will go through a set of standardized questions related to any general anxieties, stress, and inability to relax you may have been experiencing in the last 2 weeks. Respond using the following answer scale: 0 - Not at all sure, 1 - Several Days, 2 - Over half the days, or 3- Nearly Every day. Please indicate what feels most accurate for you thinking about the last 2 weeks.

1. Feeling nervous, anxious, or on edge

Not at all (0)

Several days (1)

More than half the days (2)

Nearly every day (3)

Choose not to answer

1. Not being able to stop or control worrying

Not at all (0)

Several days (1)

More than half the days (2)

Nearly every day (3)

Choose not to answer

**CAGE-AID**

1. Have you ever felt that you ought to cut down on your drinking or drug use?

Yes (1)

No (0)

Choose not to answer

1. Have people annoyed you by criticizing your drinking or drug use?

Yes (1)

No (0)

Choose not to answer

1. Have you ever felt bad or guilty about your drinking or drug use?

Yes (1)

No (0)

Choose not to answer

1. Have you ever had a drink or used drugs first thing in the morning to steady your nerves or to get rid of a hangover?

Yes (1)

No (0)

Choose not to answer

1. In the past 6 months, how often do you have six or more drinks on one occasion?

Never (1)

Less than monthly (2)

Monthly (3)

Weekly (4)

Daily or almost daily (5)

**Suicidal Behaviors**

**SBQ-R**

1. Have you ever thought about or attempted to kill yourself?

Never (1)

It was just a brief passing thought (2)

I have had a plan at least once to kill myself but did not try to do it (3)

I have had a plan at least once to kill myself and really wanted to die (3)

I have attempted to kill myself, but did not want to die (4)

I have attempted to kill myself and really hoped to die (4)

Choose not to answer

2. How often have you thought about killing yourself in the past year?

Never (1)

Rarely (1 time) (2)

Sometimes (2 times) (3)

Often (3-4 times (4)

Very Often (5 or more times) (5)

Choose not to answer

3. Have you ever told someone that you were going to commit suicide, or that you might do it?

No (1)

Yes, at one time, but did not really want to die (2)

Yes, at one time, and really wanted to die (2)

Yes, more than once, but did not really want to do it (3)

Yes, more than once, and really wanted to do it (3)

Choose not to answer

4. How likely is it that you will attempt suicide some day?

Never (0)

No chance at all (1)

Rather unlikely (2)

Unlikely (3)

Likely (4)

Rather likely (5)

Very likely (6)

Choose not to answer

*If total score on SBQ-R is equal to or greater than 7, then, “We are concerned by some of your responses and recommend that you seek help and support. You can reach out for immediate crisis help from: National Suicide Prevention Hotline (1-800-273-TALK or through chat at https://suicidepreventionlifeline.org) Crisis Textline (Text HOME to 741741 or go to https://www.crisistextline.org. If you are not in crisis but would like mental health support, please see https://mhanational.org or https://nami.org/Your-Journey.”*

**Suicide History**

Have you attempted to kill yourself?

Yes

No

**DMHT Questionnaire**

**Part 1. Use of DMHTs during COVID-19**

1. Have you downloaded or used an app to help you cope with stress associated with COVID-19? Yes or No

*If “Yes” to A., participant given the following questions:*

1. What app did you try? If you tried more than one app, please pick the one you liked the most. *(Open-response)*
2. What did you like most about the app? *(Open-response)*
3. What did you like least about the app? *(Open-response)*
4. What changes would you make to the app to improve it? *(Open-response)*

**Part 2. Usability and burden of DMHTs during COVID-19**

*If “Yes” to A., then participant received the System Usability Questionnaire and User Burden Questionnaire. Not provided in Supplemental Materials due to copyright law.*

*If “No” to A., participant given the following:*

Which of these are reasons why you haven’t used an app to cope with COVID-19? Please indicate.

| **Reason** | Checkbox |
| --- | --- |
| I didn’t think to look for an app |  |
| I couldn’t find an app that was relevant to what I needed |  |
| I don’t have time to use an app to cope |  |
| I don’t have money to spend on data plan to use apps |  |
| I don’t think apps would help me |  |
| I prefer to work with a professional |  |
| I have other ways of coping |  |
| Other reason |  |

Please list your strategies for coping: *(open-response)*

Other reason, please specify: *(open-response)*

**Part 3: Design of COVID-19 App**

There are many kinds of apps in Play and App Stores that are developed to help you with your mood, stress, and some to cope with COVID-19. The top-rated apps are somewhat different from each other. We are interested in learning about which features you feel an app to help you with the emotional consequences of COVID-19 should possess. Please rank each app feature below from not at all important to very important (0-9).

1. Information or education

| 0 | 1 | | 2 | | 3 | | | 4 | | 5 | | 6 | | 7 | | 8 | | 9 |
| --- | --- | --- | --- | --- | --- | --- | --- | --- | --- | --- | --- | --- | --- | --- | --- | --- | --- | --- |
| Not at all important | |  | |  | |  |  | |  | |  | |  | |  | | Very important | |

1. Mindfulness/meditation tools

| 0 | 1 | | 2 | | 3 | | | 4 | | 5 | | 6 | | 7 | | 8 | | 9 |
| --- | --- | --- | --- | --- | --- | --- | --- | --- | --- | --- | --- | --- | --- | --- | --- | --- | --- | --- |
| Not at all important | |  | |  | |  |  | |  | |  | |  | |  | | Very important | |

1. Symptom tracking (tracking sleep or mood)

| 0 | 1 | | 2 | | 3 | | | 4 | | 5 | | 6 | | 7 | | 8 | | 9 |
| --- | --- | --- | --- | --- | --- | --- | --- | --- | --- | --- | --- | --- | --- | --- | --- | --- | --- | --- |
| Not at all important | |  | |  | |  |  | |  | |  | |  | |  | | Very important | |

1. Brain games to improve thinking

| 0 | 1 | | 2 | | 3 | | | 4 | | 5 | | 6 | | 7 | | 8 | | 9 |
| --- | --- | --- | --- | --- | --- | --- | --- | --- | --- | --- | --- | --- | --- | --- | --- | --- | --- | --- |
| Not at all important | |  | |  | |  |  | |  | |  | |  | |  | | Very important | |

1. Distraction tools (drawing, puzzles, music)

| 0 | 1 | | 2 | | 3 | | | 4 | | 5 | | 6 | | 7 | | 8 | | 9 |
| --- | --- | --- | --- | --- | --- | --- | --- | --- | --- | --- | --- | --- | --- | --- | --- | --- | --- | --- |
| Not at all important | |  | |  | |  |  | |  | |  | |  | |  | | Very important | |

1. Tools to focus on the positive events and influences in life

| 0 | 1 | | 2 | | 3 | | | 4 | | 5 | | 6 | | 7 | | 8 | | 9 |
| --- | --- | --- | --- | --- | --- | --- | --- | --- | --- | --- | --- | --- | --- | --- | --- | --- | --- | --- |
| Not at all important | |  | |  | |  |  | |  | |  | |  | |  | | Very important | |

1. Link to resources, counseling, or crisis support

| 0 | 1 | | 2 | | 3 | | | 4 | | 5 | | 6 | | 7 | | 8 | | 9 |
| --- | --- | --- | --- | --- | --- | --- | --- | --- | --- | --- | --- | --- | --- | --- | --- | --- | --- | --- |
| Not at all important | |  | |  | |  |  | |  | |  | |  | |  | | Very important | |

1. A chatbot to help you cope with daily stress

| 0 | 1 | | 2 | | 3 | | | 4 | | 5 | | 6 | | 7 | | 8 | | 9 |
| --- | --- | --- | --- | --- | --- | --- | --- | --- | --- | --- | --- | --- | --- | --- | --- | --- | --- | --- |
| Not at all important | |  | |  | |  |  | |  | |  | |  | |  | | Very important | |

1. How to cope with COVID-19

| 0 | 1 | | 2 | | 3 | | | 4 | | 5 | | 6 | | 7 | | 8 | | 9 |
| --- | --- | --- | --- | --- | --- | --- | --- | --- | --- | --- | --- | --- | --- | --- | --- | --- | --- | --- |
| Not at all important | |  | |  | |  |  | |  | |  | |  | |  | | Very important | |

1. Other feature *(Write in):* _________________

| 0 | 1 | | 2 | | 3 | | | 4 | | 5 | | 6 | | 7 | | 8 | | 9 |
| --- | --- | --- | --- | --- | --- | --- | --- | --- | --- | --- | --- | --- | --- | --- | --- | --- | --- | --- |
| Not at all important | |  | |  | |  |  | |  | |  | |  | |  | | Very important | |

Now it’s time for you to build your own app. Which of these features above would you put in the app? Please indicate yes or no for each feature.

| **App feature** | Yes or No |
| --- | --- |
| Information or Education |  |
| Mindfulness/meditation |  |
| Symptom tracking (tracking sleep or mood) |  |
| Brain games to improve thinking |  |
| Distraction tools (drawing, puzzles, music) |  |
| Tools to focus on the positive events and influences in life |  |
| Link to resources, counseling or crisis support |  |
| A chatbot to help you with daily stress |  |
| How to cope with COVID-19 |  |

Is there anything not listed you would add to the app? *(Open-ended)*
