# Supplementary material for: Mental Health and the Perceived Usability of Digital Mental Health Tools Among Essential Workers and People Unemployed Due to COVID-19: Cross-sectional Survey Study
Source: JMIR Ment Health. 2021 Aug 5;8(8):e28360. doi: 10.2196/28360 (PMC8354319; doi:10.2196/28360)
Supplement: Multimedia Appendix 2 [file mental_v8i8e28360_app2.doc]

**Multimedia Appendix 2.** Distress Measures Stratified by App Users

|  | Non App User (n=1680) | App User (n=277) | Total (N=1957) | *P*-value |
| --- | --- | --- | --- | --- |
|  |  |  |  |  |
| **PHQ Total Score**  **(Range: 0-6)** |  |  |  | **0.02^1^** |
| Mean (*SD*) | 2.4 (1.8) | 2.6 (1.7) | 2.4 (1.8) |  |
|  |  |  |  |  |
| **PHQ-2 Interpretation**, n (%) |  |  |  | 0.07^2^ |
| No/low depression | 1064 (63.4%) | 160 (57.8%) | 1224 (62.6%) |  |
| Clinically Significant | 614 (36.6%) | 117 (42.2%) | 731 (37.4%) |  |
|  |  |  |  |  |
| **GAD-2 Total score**  **(Range: 0-6)** |  |  |  | **<.01^1^** |
| Mean (SD) | 2.6 (2.0) | 3.1 (1.9) | 2.7 (2.0) |  |
|  |  |  |  |  |
| **GAD-2 Interpretation**, n (%) |  |  |  | **<.01^2^** |
| Not/low anxiety | 926 (55.2%) | 118 (42.6%) | 1044 (53.4%) |  |
| Likely anxiety | 752 (44.8%) | 159 (57.4%) | 911 (46.6%) |  |
|  |  |  |  |  |
| **CAGE-AID Score**  **(Range: 0-4)** |  |  |  | **<.01^1^** |
| Mean (SD) | 1.0 (1.4) | 1.3 (1.5) | 1.1 (1.4) |  |
|  |  |  |  |  |
| **CAGE-AID Interpretation**, n (%) |  |  |  | **<.01^2^** |
| No SUD | 948 (57.4%) | 124 (46.1%) | 1072 (55.8%) |  |
| At risk for SUD | 704 (42.6%) | 145 (53.9%) | 849 (44.2%) |  |
|  |  |  |  |  |
| **SBQ-R Total Score**  **(Range: 1-18)** |  |  |  | **0.03^1^** |
| Mean (SD) | 6.3 (3.6) | 6.8 (3.8) | 6.4 (3.6) |  |
|  |  |  |  |  |
| **SBQ-R Interpretation**, n (%) |  |  |  | **0.04^2^** |
| Low to no risk | 1057 (63.0%) | 157 (56.7%) | 1214 (62.1%) |  |
| With risk | 621 (37.0%) | 120 (43.3%) | 741 (37.9%) |  |
|  |  |  |  |  |
| **History of Suicide Attempt,** n (%) |  |  |  | 0.26^2^ |
| No | 1396 (84.2%) | 225 (81.5%) | 1621 (83.8%) |  |
| Yes | 262 (15.8%) | 51 (18.5%) | 313 (16.2%) |  |
|  |  |  |  |  |
| **Psychological Distress**, n (%) |  |  |  | <.01^2^ |
| Non-distressed | 446 (26.5%) | 41 (14.8%) | 487 (24.9%) |  |
| Distressed | 1234 (73.5%) | 236 (85.2%) | 1470 (75.1%) |  |

^1^Equal variance two sample t-test; ^2^Chi-Square *P*-value; ^3^Unequal variance two sample t-test;

**BOLD** indicates *P*-value <.05 and less than Benjamini-Hochberg critical value, considered to be statistically significant
