# Supplementary material for: Mental Health and the Perceived Usability of Digital Mental Health Tools Among Essential Workers and People Unemployed Due to COVID-19: Cross-sectional Survey Study
Source: JMIR Ment Health. 2021 Aug 5;8(8):e28360. doi: 10.2196/28360 (PMC8354319; doi:10.2196/28360)
Supplement: Multimedia Appendix 3 [file mental_v8i8e28360_app3.docx]

## **Supplementary Materials**

**Table S1.** Distress Measures Stratified by Workers and DMHT users

|  | Unemployed    (n=1013) | Essential Worker    (n=974) | *P*-value | Non-DMHT User   (n=1680) | DMHT User   (n=277) | *P*-value | Total    (N=1987) |
| --- | --- | --- | --- | --- | --- | --- | --- |
|  |  |  |  |  |  |  |  |
| **PHQ-2 Interpretation**, n (%) |  |  | *<.01^a^* |  |  | .07 **^a^** |  |
| No/low depression | 537 (53.5%) | 700 (72.2%) |  | 1064 (63.4%) | 160 (57.8%) |  | 1237 (62.7%) |
| Clinically Significant | 467 (46.5%) | 270 (27.8%) |  | 614 (36.6%) | 117 (42.2%) |  | 737 (37.3%) |
|  |  |  |  |  |  |  |  |
| **GAD-2 Interpretation**, n (%) |  |  | *<.01 ^a^* |  |  | *<.01 ^a^* |  |
| Not/low anxiety | 445 (44.3%) | 611 (63.0%) |  | 926 (55.2%) | 118 (42.6%) |  | 1056 (53.5%) |
| Likely anxiety | 559 (55.7%) | 359 (37.0%) |  | 752 (44.8%) | 159 (57.4%) |  | 918 (46.5%) |
|  |  |  |  |  |  |  |  |
| **CAGE-AID Interpretation**, n (%) |  |  | *<.01^a^* |  |  | *<.01 ^a^* |  |
| No SUD | 484 (49.3%) | 598 (62.7%) |  | 948 (57.4%) | 124 (46.1%) |  | 1082 (55.9%) |
| Possible SUD | 498 (50.7%) | 356 (37.3%) |  | 704 (42.6%) | 145 (53.9%) |  | 854 (44.1%) |
|  |  |  |  |  |  |  |  |
| **SBQ-R Interpretation**, n (%) |  |  | *<.01 ^a^* |  |  | *.04 ^a^* |  |
| Low to no risk | 544 (54.7%) | 672 (69.9%) |  | 1057 (63.0%) | 157 (56.7%) |  | 1216 (62.1%) |
| With risk | 451 (45.3%) | 290 (30.1%) |  | 621 (37.0%) | 120 (43.3%) |  | 741 (37.9%) |
|  |  |  |  |  |  |  |  |
| **History of Suicide Attempt,** n (%) |  |  | *<.01 ^a^* |  |  | .26 **^a^** |  |
| No | 794 (80.9%) | 829 (86.9%) |  | 1396 (84.2%) | 225 (81.5%) |  | 1623 (83.8%) |
| Yes | 188 (19.1%) | 125 (13.1%) |  | 262 (15.8%) | 51 (18.5%) |  | 313 (16.2%) |
|  |  |  |  |  |  |  |  |
| **Psychological Distress**, n (%) |  |  | *<.01 ^a^* |  |  | *<.01 ^a^* |  |
| Non-distressed | 189 (18.8%) | 308 (31.7%) |  | 446 (26.5%) | 41 (14.8%) |  | 497 (25.2%) |
| Distressed | 815 (81.2%) | 664 (68.3%) |  | 1234 (73.5%) | 236 (85.2%) |  | 1479 (74.8%) |
|  |  |  |  |  |  |  |  |

^a^  Chi-Square *P*-value

*Italics* indicates *P*-value <.05 **and** less than Benjamini-Hochberg critical value, considered to be statistically significant.

**Table S2.** App Categories Stratified by Worker and Psychological Distress

|  | Unemployed  (n=131) | Essential Worker  (n=130) | *P*-value | Non-distressed  (n=38) | Distressed  (n=223) | *P*-value | Total  (N=261) |
| --- | --- | --- | --- | --- | --- | --- | --- |
|  |  |  |  |  |  |  |  |
| **App Categories**, n (%) |  |  | *<.01^a^* |  |  | .67^a^ |  |
| Meditation/Mindfulness | 70 (53.4%) | 49 (37.7%) |  | 19 (50.0%) | 100 (44.8%) |  | 119 (45.6%) |
| Virtual Therapy or Contact with Virtual Provider | 11 (8.4%) | 14 (10.8%) |  | 1 (2.6%) | 24 (10.8%) |  | 25 (9.6%) |
| Chat Feature | 11 (8.4%) | 10 (7.7%) |  | 2 (5.3%) | 19 (8.5%) |  | 21 (8.0%) |
| Health | 8 (6.1%) | 12 (9.2%) |  | 2 (5.3%) | 18 (8.1%) |  | 20 (7.7%) |
| COVID-19 Contact Tracing | 1 (0.8%) | 12 (9.2%) |  | 4 (10.5%) | 9 (4.0%) |  | 13 (5.0%) |
| Entertainment and Distraction | 8 (6.1%) | 4 (3.1%) |  | 2 (5.3%) | 10 (4.5%) |  | 12 (4.6%) |
| Social Media | 4 (3.1%) | 6 (4.6%) |  | 3 (7.9%) | 7 (3.1%) |  | 10 (3.8%) |
| Symptom Track | 7 (5.3%) | 3 (2.3%) |  | 1 (2.6%) | 9 (4.0%) |  | 10 (3.8%) |
| COVID-19 Coping | 0 (0.0%) | 8 (6.2%) |  | 2 (5.3%) | 6 (2.7%) |  | 8 (3.1%) |
| Finance | 0 (0.0%) | 7 (5.4%) |  | 0 (0.0%) | 7 (3.1%) |  | 7 (2.7%) |
| Positive Psychology | 5 (3.8%) | 2 (1.5%) |  | 1 (2.6%) | 6 (2.7%) |  | 7 (2.7%) |
| Journal | 3 (2.3%) | 1 (0.8%) |  | 1 (2.6%) | 3 (1.3%) |  | 4 (1.5%) |
| News | 1 (0.8%) | 2 (1.5%) |  | 0 (0.0%) | 3 (1.3%) |  | 3 (1.1%) |
| Crisis | 1 (0.8%) | 0 (0.0%) |  | 0 (0.0%) | 1 (0.4%) |  | 1 (0.4%) |
| Language Learning | 1 (0.8%) | 0 (0.0%) |  | 0 (0.0%) | 1 (0.4%) |  | 1 (0.4%) |
|  |  |  |  |  |  |  |  |

^a^  Chi-Square *P*-value

*Italics* indicates *P*-value <.05 **and** less than Benjamini-Hochberg critical value, considered to be statistically significant.

**Table S3.** Lack of DMHT use stratified by Workers and Psychological Distress

|  | Unemployed   (n*=*876) | Essential Worker   (n*=*834) | *P-*value | Non-distressed  (n=456) | Distressed  (n=1243) | *P*-value | Total   (N=1710) |
| --- | --- | --- | --- | --- | --- | --- | --- |
|  |  |  |  |  |  |  |  |
| **Reasons why you haven't used an app to cope with COVID** |  |  |  |  |  |  |  |
| I didn’t think to look for an app | 629 (71.8%) | 550 (65.9%) | *<.01^a^* | 293 (64.3%) | 886 (71.3%) | *<.01^a^* | 1179 (68.9%) |
| I don’t think apps would help me | 324 (37.0%) | 281 (33.7%) | .15^a^ | 142 (31.1%) | 463 (37.2%) | *.02^a^* | 605 (35.4%) |
| I have others  ways of coping | 232 (26.5%) | 189 (22.7%) | .07^a^ | 140 (30.7%) | 281 (22.6%) | *<.01^a^* | 421 (24.6%) |
| I prefer to work with a profess-ional | 124 (14.2%) | 99 (11.9%) | .16^a^ | 32 (7.0%) | 191 (15.4%) | *<.01^a^* | 223 (13.0%) |
| I don’t have time to use an app to cope | 84 (9.6%) | 94 (11.3%) | .25^a^ | 39 (8.6%) | 139 (11.2%) | .12^a^ | 178 (10.4%) |
| I don’t have money to spend on a data plan to use apps | 112 (12.8%) | 54 (6.5%) | *<.01^a^* | 25 (5.5%) | 141 (11.3%) | *<.01^a^* | 166 (9.7%) |
| I couldn’t find an app that was  relevant to what I needed | 66 (7.5%) | 56 (6.7%) | .51^a^ | 19 (4.2%) | 103 (8.3%) | *<.01^a^* | 122 (7.1%) |
| Other | 31 (3.5%) | 37 (4.4%) | .34^a^ | 21 (4.6%) | 47 (3.8%) | .44^a^ | 68 (4.0%) |
|  |  |  |  |  |  |  |  |

^a^Chi-Square *P*-value

*Italics* indicates *P*-value <.05 **and** less than Benjamini-Hochberg critical value, considered to be statistically significant. Multiple responses were permitted; total adds to more than 100%.

**Table S4.**User Burden & System Usability Stratified by Top 3 Apps

|  | Calm    (n=41) | Headspace    (n=38) | BetterHelp    (n=11) | Total    (N=90) | *P*-value |
| --- | --- | --- | --- | --- | --- |
|  |  |  |  |  |  |
| **Difficulty of Use** |  |  |  |  | .75^a^ |
| N | 41 | 38 | 11 | 90 |  |
| Mean (SD) | 1.7 (2.8) | 1.4 (1.9) | 1.2 (1.3) | 1.5 (2.3) |  |
| Range | 0.0, 14.0 | 0.0, 7.0 | 0.0, 4.0 | 0.0, 14.0 |  |
|  |  |  |  |  |  |
| **Physical Burden** |  |  |  |  | .29^a^ |
| N | 41 | 38 | 11 | 90 |  |
| Mean (SD) | 0.5 (1.9) | 0.1 (0.4) | 0.0 (0.0) | 0.3 (1.3) |  |
| Range | 0.0, 11.0 | 0.0, 2.0 | 0.0, 0.0 | 0.0, 11.0 |  |
|  |  |  |  |  |  |
| **Social & Time Burden** |  |  |  |  | .42 ^a^ |
| N | 41 | 38 | 11 | 90 |  |
| Mean (SD) | 1.1 (2.9) | 0.5 (0.9) | 0.7 (1.2) | 0.8 (2.1) |  |
| Range | 0.0, 15.0 | 0.0, 4.0 | 0.0, 4.0 | 0.0, 15.0 |  |
|  |  |  |  |  |  |
| **Mental & Emotional Burden** |  |  |  |  | .07^a^ |
| N | 41 | 38 | 11 | 90 |  |
| Mean (SD) | 1.5 (3.0) | 0.3 (0.8) | 0.7 (1.1) | 0.9 (2.2) |  |
| Range | 0.0, 14.0 | 0.0, 3.0 | 0.0, 3.0 | 0.0, 14.0 |  |
|  |  |  |  |  |  |
| **Privacy Burden** |  |  |  |  | *.04 ^a^* |
| N | 41 | 38 | 11 | 90 |  |
| Mean (SD) | 1.5 (2.8) | 0.5 (1.0) | 2.0 (2.1) | 1.2 (2.2) |  |
| Range | 0.0, 10.0 | 0.0, 4.0 | 0.0, 6.0 | 0.0, 10.0 |  |
|  |  |  |  |  |  |
| **Financial Burden** |  |  |  |  | .12^1a^ |
| N | 41 | 38 | 11 | 90 |  |
| Mean (SD) | 3.4 (2.6) | 2.6 (2.4) | 4.3 (2.4) | 3.2 (2.5) |  |
| Range | 0.0, 8.0 | 0.0, 8.0 | 0.0, 8.0 | 0.0, 8.0 |  |
|  |  |  |  |  |  |
| **System Usability Score** |  |  |  |  | .21 ^a^ |
| N | 41 | 38 | 11 | 90 |  |
| Mean (SD) | 76.2 (17.7) | 81.3 (13.4) | 83.4 (8.5) | 79.2 (15.2) |  |
| Range | 20.0, 97.5 | 42.5, 100.0 | 65.0, 92.5 | 20.0, 100.0 |  |
|  |  |  |  |  |  |

^a^ANOVA F-test p-value;

*Italics* indicates *P*-value <.05 and less than Benjamini-Hochberg critical value, considered to be statistically significant
